# Reduction of Subcutaneous and Visceral Fat With the Use of Energy‐Based Equipment With a High‐Power Amplifying Effect Plus an Exercise Regimen

**DOI:** 10.1111/jocd.70490

**Published:** 2025-10-08

**Authors:** Jesús Rodriguez Lastra, Sidra Kouser

**Affiliations:** ^1^ Universidad Alfonso X El Sabio Madrid Spain; ^2^ Clinical Researcher & Physiotherapist INNEO Clinic Barcelona Barcelona Spain

**Keywords:** abdominal fat loss, amplified radiofrequency, Capenergy, exercise, subcutaneous fat loss, visceral fat loss

## Abstract

**Background:**

The use of electromagnetic energy as an alternative to surgical procedures for subcutaneous fat reduction has gained popularity in recent years. In this pilot study, the effects of using amplified radio‐frequency energy in the medium wave delivery range, in conjunction with exercise intervention, were investigated for their potential reduction of abdominal subcutaneous and visceral fat.

**Objectives:**

The study aimed to evaluate the effects of combined intervention on the volume of abdominal fat, analyzing changes in lipid profile, metabolic factors, and inflammatory markers.

**Methods:**

Thirty‐four participants were selected and assigned to a 10‐day intervention utilizing a Capenergy device every weekday except Saturday and Sunday. This involved the application of amplified energy through a belt covering the abdominal area of 800 cm^2^ followed by a 45‐min submaximal aerobic exercise. Measurements were conducted pretreatment and after the intervention. Lipid profile, leptin, insulin, and protein C were measured. Body fat was measured by MRI of the entire abdomen. Follow‐up measurements were also taken at the 6‐month mark.

**Results:**

A statistically significant decrease was noted in both subcutaneous and visceral fat in the abdominal area, as well as a decrease in weight, waist‐to‐hip, and all biochemical values, without side effects, just a slight redness. These effects on abdominal fat were maintained at the 6‐month follow‐up with no reports of regaining fat.

**Conclusion:**

The utilization of amplified radiofrequency with an abdominal belt with static electrodes and temperature sensors, together with exercise intervention, has significant potential for reducing abdominal subcutaneous and visceral fat, while also improving associated factors safely and effectively, even after a follow‐up period.

**Trial Registration:** NCT06377358

## 1 Introduction

Subcutaneous fat loss and body contouring are some of the most pressing concerns of body aesthetics. Many patients do not want to undergo surgical treatment because of the painful recovery, the postoperative time that separates them from daily activities, and the fear of being subjected to an invasive procedure. An alternative to this procedure is the use of electromagnetic energy, one of which is in the medium wave delivery range, between 0.5 and 3 MHz approximately, also known as “radio waves” or “radio frequency”, of which there are several publications [1, 2, 3]. Some authors have combined radiofrequency with electrical stimulation of muscle contraction [4, 5]. The results obtained from initial investigations primarily concern decreases in waist circumference measured in centimeters [6]. In other studies, magnetic resonance imaging (MRI) has been used to quantify the amount of fat present in the abdomen, through the slice of the image at the L2–L3 level of the intervertebral space with a thickness of 5 cm, which is then generalized to the total volume of the abdomen.

However, it has raised concerns regarding the reliability of using a single MRI slice to estimate amounts of visceral adipose tissue (VAT) and subcutaneous adipose tissue (SAT). It has been noted that the overall measurement of fat obtained by this method may not be precise due to variations in the height of the MRI section resulting from weight loss, as fat distribution in the abdominal region can vary substantially. Consequently, in specific study designs, the use of multiple MRI slices has been deemed a more robust approach for identifying alterations in VAT and SAT compared to relying on a single slice [7]. There are four methods of measuring abdominal fat: ultrasound, DEXA, CT, and MRI. The latter two (CT and MRI) are considered reference methods for measuring interstitial and subcutaneous adipose tissue in vivo [8]. The utilization of MRI techniques, as compared to CT scans, offers the inherent benefit of not subjecting the patient to ionizing radiation, thus promoting greater overall safety.

The effect of Radiofrequency energy on adipose tissue is based on the heating of the tissue, which liquefies the fat inside the adipocyte, dissolving it, and generating pores in its membrane [9] that release fat, with consecutive apoptosis [10]. To achieve this effect, three elements are needed: first, a frequency of 1 MHz, which has been pointed out as the one that acts best with the adipocyte membrane, and which allows the energy to be delivered to the exact area where the fat is located. Second, a larger surface area of application to introduce more energy, and third, high power.

A concern raised by this treatment is to identify the final destination of the triglycerides that are mobilized in the procedure with this equipment, based on amplified electromagnetic energy. The catabolism of triglycerides, or their conversion into energy, may yield positive impacts for patients beyond the intended lipolysis. Submaximal exercise is recognized to rely predominantly on lipid utilization to generate energy, derived from the accumulation of fatty acids (FAs) in the SAT and intramuscular and dietary fat stores. Maximal oxidation of FAs occurs mainly when exercise intensity is between 45% and 65% of VO_2_ max.

One potential approach to address the outcome of radiofrequency‐induced triglyceride mobilization is through submaximal intensity exercise, which would prompt the utilization of adipocyte‐released triglycerides by muscles. A research study involving both exercise and radiofrequency treatments demonstrated substantial reductions in waist circumference, SAT, as measured by ultrasound, and abdominal fold among female participants following four exercise sessions [11]. The reported findings indicate a reduction in both the waist‐to‐height ratio and waist circumference; however, these changes were presented without assessing pertinent biochemical indicators of the lipid profile or examination of potential alterations in adipose tissue, inflammation, insulin, and leptin levels. It is noteworthy that leptin is primarily secreted by adipose tissue, but also by other body tissues, including the stomach, placenta, and mammary gland.

We conducted a pilot study to evaluate the effects of combined wide‐area amplified radiofrequency, where we hypothesize that constructive interference waves align in phase such that their amplitudes add up, potentially creating localized regions of higher energy density, producing the cross‐linking action [12] of high‐power electromagnetic beams on abdominal subcutaneous and visceral fat, which translates into high fat loss. Abdominal fat volume was quantified using MRI, while secondary analyses included assessments of lipid profiles, metabolic parameters, adipocyte‐related biomarkers, and inflammatory markers such as C‐reactive protein (CRP). Submaximal physical exercise was incorporated immediately after each treatment session to facilitate the metabolism of lipids released by adipocytes. Moderate exercise was added to achieve the consumption of triglycerides that are mobilized in the body.

### 1.1 Methodology

The sample size was determined through the utilization of EPINFO software. The study featured a total of 30 participants. An additional four subjects were incorporated to anticipate potential attrition during the implementation of the intervention. This study involved a cohort of 34 participants between the ages of 31 and 59, for a median age of 47.2, including an even distribution of 17 women and 17 men. Participants were chosen based on specific criteria, such as a Waist‐Hip Index above 0.84 for women and 0.94 for men, a body mass index (BMI) indicating overweight (25–29.9) or obesity (≥ 30 kg/m^2^), and informed consent was obtained from all participants. Any individuals with contra‐indications to radiofrequency treatment, such as pacemakers, metal implants, active infections, a history of cancer or previous treatments, chemotherapy, radiotherapy, or diabetes or metabolic disease, were excluded from the study.

Each subject's predicted maximum heart rate (HR) was calculated, which was specified according to the equation proposed by Tanaka (HR_max_ = 208–0.7 × Age). Based on these values, Karvonen's equation was used, the THR (TFC = rest dHR + Intensity × Recovery Factor Coefficient (RFC)), and the training intensity was calculated for each participant, considering the intensity of 45%–55% of the resting heart rate (RHR), (Heart Rate Reserve (HRR) = HRmax − HRrest).

The determination of both resting HR and during exercise was performed with a Polar OH1 + and after sitting for 5–10 min.

Patients were asked not to follow any special diet or start exercising during treatment or for a time after posttreatment evaluation. They did not use any weight loss medications. Participants were instructed to maintain their pretreatment lifestyle throughout the study to minimize confounding variables and ensure that observed effects could be attributed to the intervention.

### 1.2 Laboratory Analysis

The treatment onset in Barcelona involved the extraction of blood samples conducted at a Certified Clinical Laboratory. The panel of investigations was performed, including triglycerides, cholesterol, high‐density lipoprotein (HDL), low‐density lipoprotein (LDL), very‐low‐density lipoprotein (VLDL), and CRP, glucose, insulin, and leptin; and an MRI was used to determine the overall abdominal fat volume, that is, VAT and SAT in cm [3] and grams. Waist and hip circumference measurements and BMI calculations were obtained using anthropometric tape. The assessments, as mentioned above, were then repeated upon completion of the treatment.

## 2 Treatment Device

**FIGURE 1:**
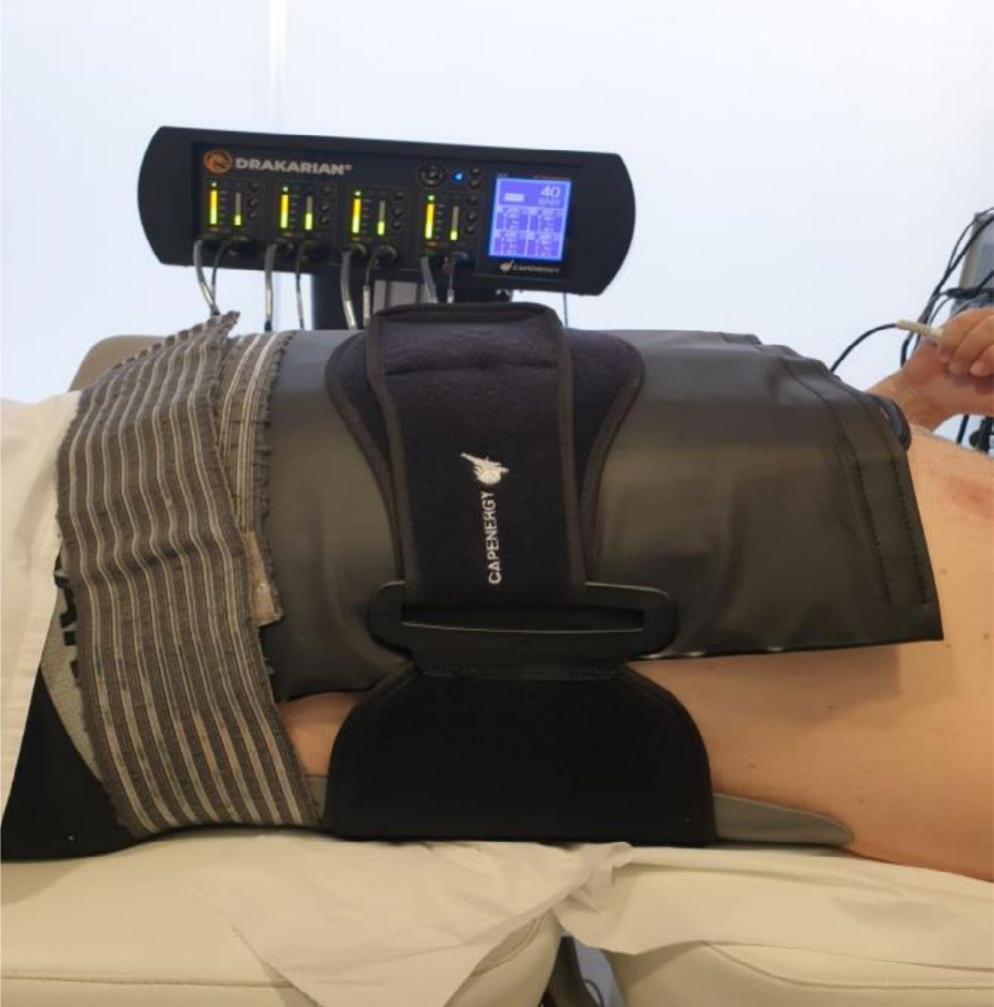
Patient equipped with Drakarian Capenergy device.

We utilized a Capenergy Medical SL Drakarian device with a power output of 1240 W, equipped with a belt‐shaped accessory designed to adapt to varying subject morphologies. The belt measured 49 cm in length and 30.5 cm in width, incorporating four plates. The total surface area of the energy‐emitting electro‐terminals was 800 cm^2^, matching that of the receiving electro‐terminals (Figure 1). The power delivery was made for 30 min, controlling the temperature through four sensors integrated into the belt. The temperature was maintained above 45°C through four integrated sensor plates in the belt, which monitored and adjusted the thermal conditions in real‐time. The study subjects reported a comfortable sensation of heat, becoming intense in some cases, but without producing burns or discomfort in any case. Five sessions were held, from Monday and Friday, for 2 weeks, for a total of 10 sessions. A slight redness of the skin was observed in some patients, which disappeared after 20 min.

Six months after the treatment, 10 patients were recruited to follow up on the procedure.

## 3 Exercise Intervention

After applying the device to mobilize abdominal fat in each patient, the Borg scale was used, and participants were asked to maintain a gentle and mildly strenuous intensity of exercise.

The procedure entailed subjecting the participants to a treadmill exercise at a 10% incline for 45 min. This was initiated with a 3‐min warm‐up at 50 W power, followed by a gradual escalation of both speed and inclination until the individual reached the target heart rate (THR). Subsequently, the subject walked for 39 min at a pace aligned with the predetermined THR range. During the 42nd–45th minutes, the power and speed were systematically lowered to induce a progressive decline in heart rate while soliciting feedback from the subject regarding their perceived level of exertion [13].

## 4 MRI Imaging

MRI scans were performed with General Electric's 3 T Sigma Pioneer MRI equipment (CETIR, Ascires Biomedical Group, Barcelona, Spain) with a Body 36/2 matrix receiving coil. Anatomical images of the entire abdomen were taken with DIXON sequences [14], and 15‐s breath holding. The axial volume was acquired, and 160 slices (3 mm each) were reconstructed. Data from each 2‐point DIXON sequence were reconstructed in the MRI system using the standard spectral model of available single‐peaked fat. Each sequence produced four images per slice: water, fat, in‐phase, and out‐of‐phase. For the volume of SAT and VAT, a volumetric segmentation of all fat from the hepatic region to the iliac crests was performed. The ITK‐SNAP software was used to obtain the measurement. This software has an accuracy with an average error of 4.7% ± 4.3% and an intra‐ and inter‐examiner reliability with intraclass correlation coefficients (ICC) > 0.99, indicating near‐perfect agreement between different users [15]. Manually, without automatic interpolation and reviewing cut by cut, visceral fat was measured separately from subcutaneous fat. The images were calculated by a biomedical engineer and analyzed by two radiologists. The Kappa coefficient, presented in Table 1, indicates an acceptable level of agreement among radiologists. This value, in relation to MRI evaluations, although not ideal, is within the range of 0.40 reported in the medical literature [16].

**TABLE 1:** Kappa coefficient showing the level of agreement among radiologists.

Symmetrical measurements					
		Value	Error típ. asint (a)	T aproximad	Approximate sig.
Measure of agreement	Kappa	0.476	0.044	4.472	0.000
No. of valid cases		34			

Each patient underwent an Inbody before and after treatment using a Lookin'Body (InBody 120) V 1.3.

## 5 Statistical Analysis

The analysis of all statistical results was carried out with the SPSS program V 26. The Kolmogorov–Smirnov test was used as a means of assessing the normality of variable values. Only leptin and insulin were not normally distributed and were analyzed with nonparametric tests; the Wilcoxon test was used to determine statistical differences. In normal distribution, a pretreatment/posttreatment comparison of mean differences was conducted using the Student's *t*‐test. Similarly, the selection of the Friedman test was appropriate in investigating occurrences of statistical significance during the 3‐month follow‐up period. A significance level of < 0.05 was used in these analyses, and statistical analysis was carried out via SPSS version 24. Graphs were generated using GraphPad Prism version 10.

## 6 Results

The results of anthropometric measurements, lipid profile, glucose, insulin, leptin, and CRP as markers of inflammation before and after treatment, as well as values in cm^3^, as in g of VAT and SAT, are presented in Table 2. It can be observed that all parameters decrease after 10 days of treatment with the procedure combined with exercise.

**TABLE 2:** Pre and post characteristics of the participants.

	Minimum	Maximum	Mean	SD
Age (years)	31.00	59.00	47.23	7.32
Height (cm)	151.00	187.00	169.29	8.44
Weight before (kg)	70.40	131.10	93.94	15.64
Weight after (kg)	67.50	127.10	91.87	15.25
Waist‐hip index before	0.74	1.07	0.89	0.082
Waist‐hip index after	0.70	1.00	0.87	0.08
VAT before (cm^3^)	1417	8622.93	3896,59	1566.83
VAT after (cm^3^)	1306.98	8526.91	3565.90	1484.25
VAT before (g)	1275.29	7766.94	3506.93	1410.15
VAT after (g)	1176.28	7674.22	3119.31	1335.82
SAT before (cm^3^)	2083.64	13955.32	6860.06	2673.24
SAT after (cm^3^)	1918.65	13692.01	6207.66	2500.15
SAT before (g)	1875.28	12559.79	6174.06	2405.91
SAT after (g)	1726.79	12322.81	5586.90	2250.13
Cholesterol before (mg/dL)	140.00	298.00	200.94	35.28
Cholesterol after (mg/dL)	135.00	296.00	187.11	36.62
Triglycerides before (mg/dL)	31.00	411.00	141.14	82.63
Triglycerides after (mg/dL)	31.00	228.00	89.85	42.31
HDL before (mg/dL)	32.00	96.00	49.5882	13.75350
HDL after (mg/dL)	27.00	100.00	48.4118	15.58383
LDL before (mg/dL)	62.00	199.00	123.2727	30.72791
LDL after (mg/dL)	78.00	212.00	120.7353	32.52440
VLDL before (mg/dL)	6.00	79.00	26.5455	13.72746
VLDL after (mg/dL)	6.00	46.00	17.9706	8.54040
Leptin before (ng/mL)	1.91	63.10	12.6662	11.45553
Leptin after (ng/mL)	1.82	28.60	9.0871	6.90247
CRP before (mg/dL)	0.29	10.01	2.9521	2.43356
CRP after (mg/dL)	0.22	10.20	3.2274	2.99731
Insulin before (mIU/L)	4.34	39.30	14.6418	8.90987
Insulin after (mIU/L)	2.77	23.20	9.7806	5.39159
Glucose before (m/mL)	85.00	129.00	97.2647	8.63856
Glucose after (mg/mL)	76.00	135.00	93.7353	10.72327

Abbreviations: CRP, C‐reactive protein; HDL, high‐density lipoprotein; LDL, low‐density lipoprotein; SAT, subcutaneous abdominal tissue; VAT, visceral abdominal tissue; VLDL, very low‐density lipoprotein.

To determine if the values obtained are normally distributed, a Kolmogorov–Smirnov test was applied with values of *p* = 0.200 except for leptin and insulin, that is, *p* = 0.21 and *p* = 0.02; CI = 95% [1.17–6.3], respectively. Table 3 shows the mean differences, where we find the statistically significant t‐test, and the Wilcoxon test shows that the decrease in values is statistically significant for leptin and insulin. Statistical analysis using the Friedman test was performed after 6 months of the intervention to examine the outcome differences between the SAT and VAT loss data.

**TABLE 3:** Difference between characteristics postintervention. The difference was calculated before—after.

	Mean difference	SD	*p*
Weight before and after	−2.07	1.2	0.001
Waist‐hip index before and after	−0.02	0.050	0.083
VAT before and after (cm^3^)	−330.69	432,6	0.001
VAT before and after (g)	−387.62	389.3	0.001
SAT before and after (cm^3^)	−652.4	599.35	0.001
SAT before and after (g)	−587.16	539.42	0.001
Cholesterol before and after (mg/dL)	−13.83	19.36	0.001
Triglycerides before and after (mg/dL)	−51.29	51.39	0.001
HDL before and after (mg/dL)	−1.17	4.94	0.175
LDL before and after (mg/dL)	−2.53	15.86	0.224
VLDL before and after (mg/dL)	−8.57	9.37	0.001
Leptin before and after (ng/mL)	−3.57	7.02	0.002
CRP after and before (mg/dL)	−0.275	2.02	0.435
Insulin before and after (mIU/L)	−4.86	6.30	0.001
Glucose before and after mg/dL	−3.52	6.83	0.005

Abbreviations: CRP, C‐reactive protein; HDL, high‐density lipoprotein; LDL, low‐density lipoprotein; SAT, subcutaneous abdominal tissue; VAT, visceral abdominal tissue; VLDL, very low‐density lipoprotein.

Table 2 presents the results of the Student's *t*‐test for comparison of pre and posttreatment results. Note that all differences resulting in a decrease from baseline were statistically significant. The Wilcoxon test for leptin and insulin is also presented, which was also found to be statistically significant.

**FIGURE 2:**
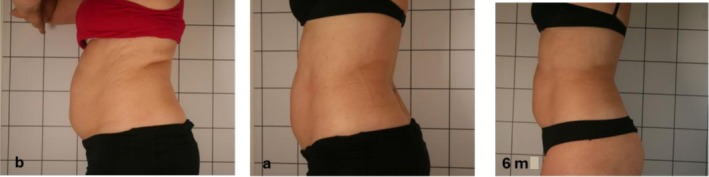
Changes in the female's body appearance from the beginning (b) to 10 days (a) and after 6 months (6 m).

**FIGURE 3:**
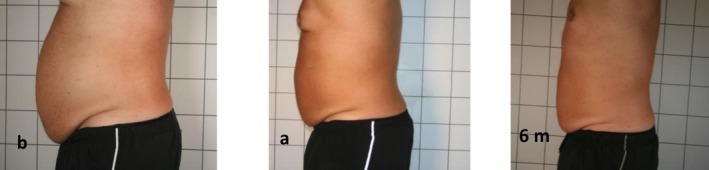
Changes in male's body appearance from the beginning (b) to 10 days (a) and after 6 months (6 m).

Figures 2 and 3 represent the alterations in the appearance of the abdomen of a female and a male, respectively, at the first and 10th sessions and after a follow‐up period of 6 months.

Figure 2 shows noticeable alterations in the physical appearance of a woman's abdomen. After undergoing 10 treatment sessions, she was able to significantly decrease her total abdominal fat (SAT and VAT), going from 7727.47 g (b) to 7136.24 g (a). She further achieved a decrease to 5078.44 g after a 6‐month (6 m) follow‐up period.

Figure 3 shows noticeable alterations in the abdominal appearance of a man, specifically in terms of a marked decrease in total abdominal fat (SAT and VAT). The total abdominal fat, initially measured at 9671.52 g (b), reduced to 8269.30 g (a) after 10 treatment sessions and further decreased to 7715.28 g (6 m) in a 6‐month follow‐up period. The reduction in male breast size observed in Figure 3 is likely due to localized fat loss induced by noninvasive radiofrequency (RF) therapy. RF energy stimulates adipocytes, promoting lipolysis and leading to fat reduction, including in the breast tissue.

**Graph 1:**
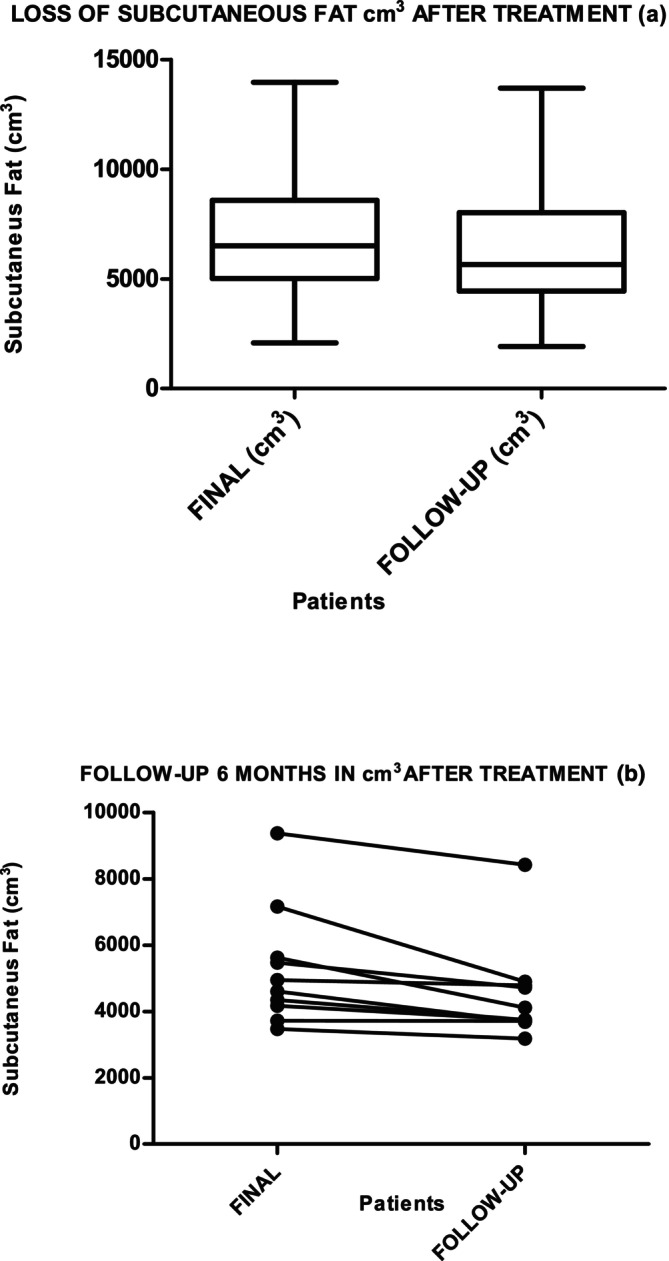
Subcutaneous fat loss (cm^3^) measured by MRI after 10 days (a) of RF and exercise treatment (*p* = 0.001 [422.3–864.6]) and (b) at 6‐month follow‐up without lifestyle modifications (*p* = 0.05).

**Graph 2:**
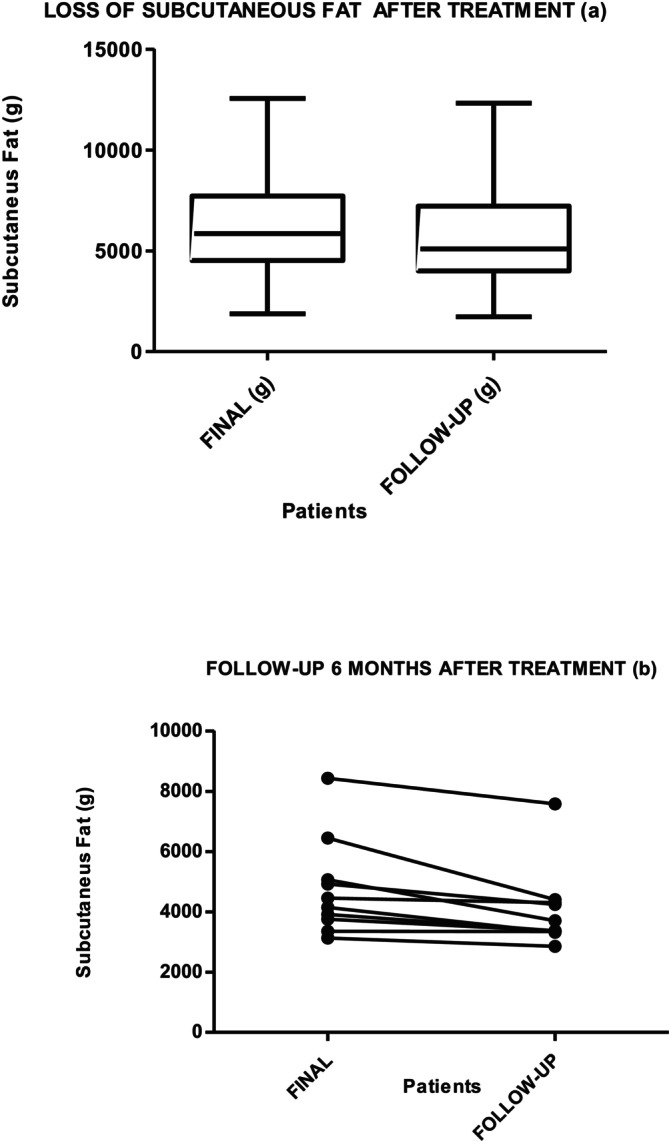
Subcutaneous fat loss (g) measured by MRI after 10 days of RF (a) and exercise treatment (*p* = 0.001 IC = 95% [380–769.2]) and (b) at 6‐month follow‐up without lifestyle modifications (*p* = 0.005).

Graphs 1 and 2 show the results of the measurement of SAT in cm^3^ and grams, at the beginning, after 10 days in 34 patients, and then in 10 participants at follow‐up at 6 months, without any change in habits: no exercise, no diet. The Friedman test for all three measures showed that the decrease maintained over time is statistically significant (*p* = 0.001). The Wilcoxon test for session 10 and the evolutionary test at 6 months showed a decrease of 789.46 cm^3^ (*p* = 0.05), with a loss of 710.52 g (*p* = 0.005). Both results are statistically significant. HDL and LDL do not show statistically significant differences.

**Graph 3:**
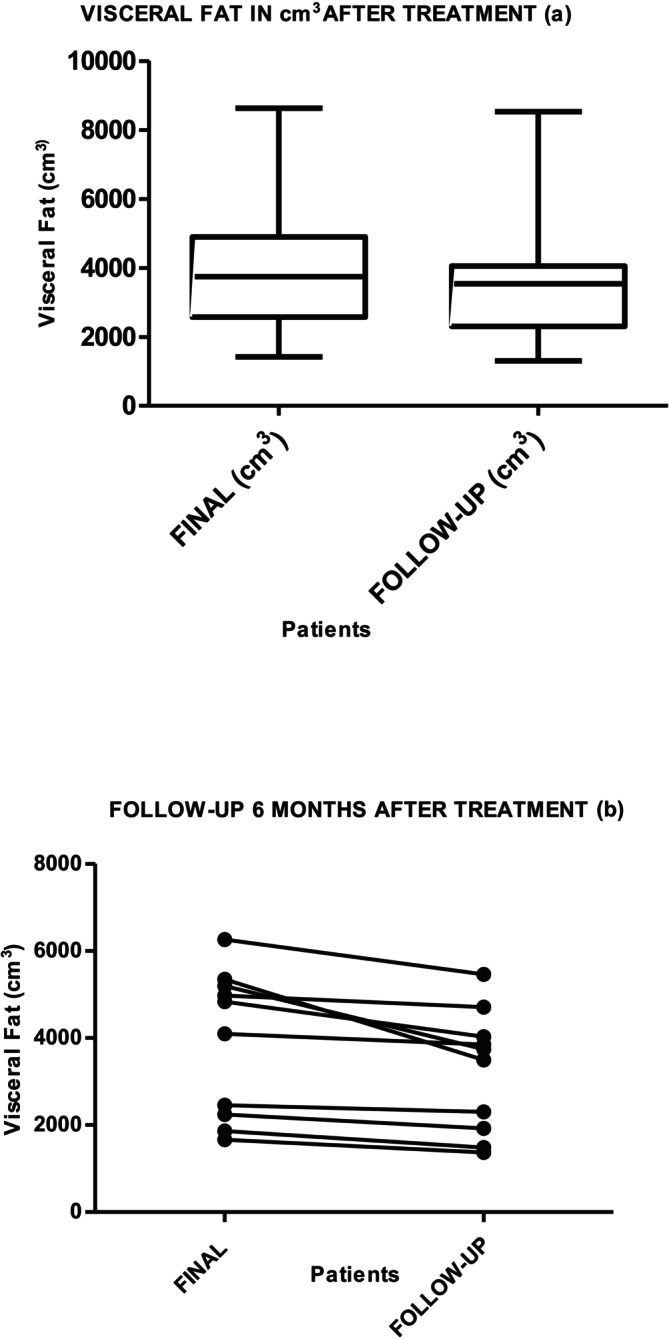
Visceral fat loss (cm^3^) measured by MRI after 10 days of radiofrequency (a) and exercise treatment (*p* = 0.001 IC = 95% [274.7–586.6]) and (b) at 6‐month follow‐up (*p* = 0.005).

**Graph 4:**
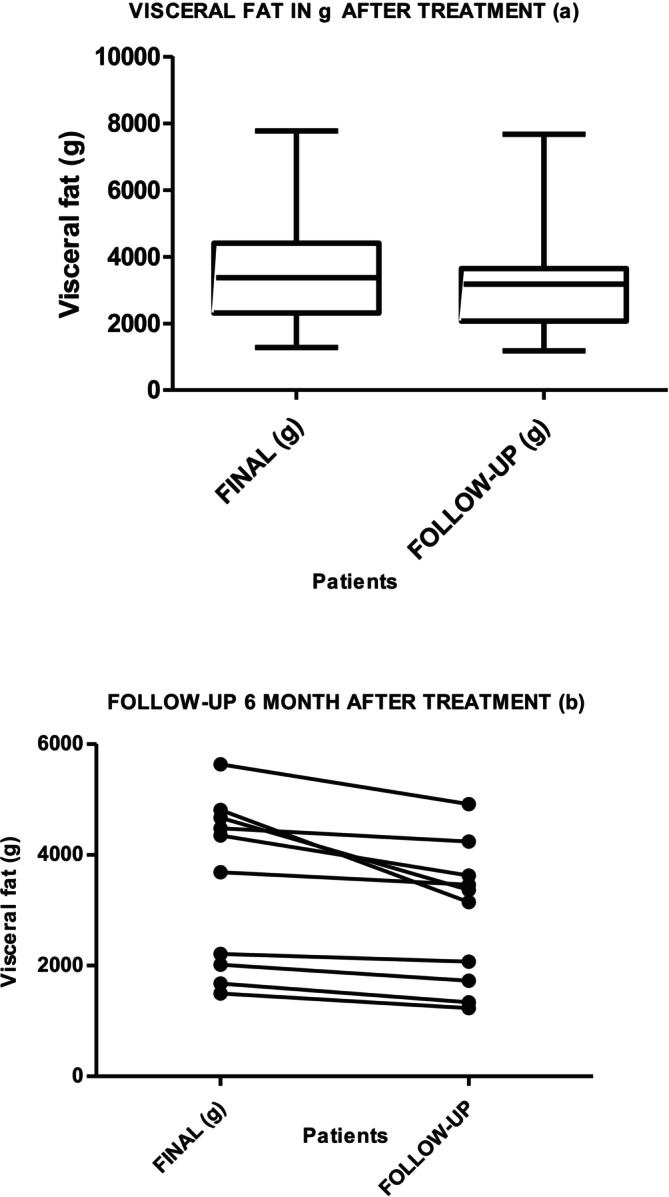
Visceral fat loss (g) measured by MRI after 10 days of radiofrequency (a) and exercise treatment (*p* = 0.001 IC = 95% [247.2–528]) and (b) at 6‐month follow‐up (*p* = 0.005).

Graphs 3 and 4 show the results of the measurement of VAT in cm^3^ and grams, at the beginning, after 10 days in 34 patients, and then in 10 participants at follow‐up at 6 months, without any change in habits: no exercise, no diet. The Friedman test for all three measurements showed that the decrease maintained over time is statistically significant (*p* = 0.001). The Wilcoxon test for session 10 and the evolutionary test at 6 months showed a decrease of 322.53 cm³ (*p* = 0.05), with a loss of 284.25 g (*p* = 0.005) in grams. Both results are statistically significant.

**FIGURE 4:**
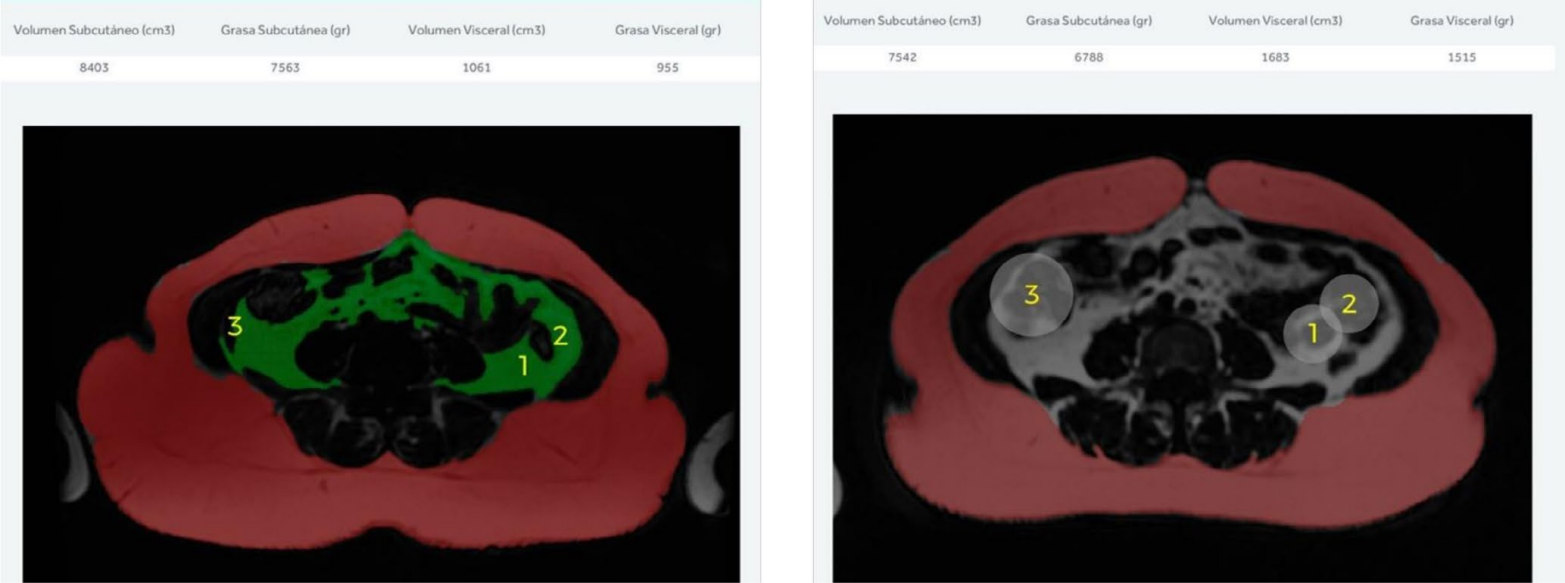
Cross‐sectional image of a male abdomen showing observed fat loss, with a reduction of 104 g in subcutaneous fat and 1312 g in visceral fat. The points of greatest loss have been pointed out. The left figure indicates before and the right indicates after.

**FIGURE 5:**
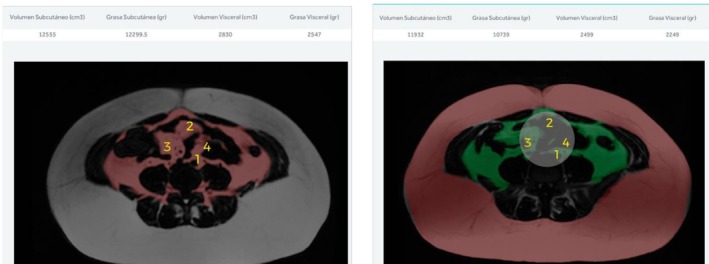
Cross‐sectional MRI image of a woman's abdomen showing observed fat loss, with a reduction of 1816 g in subcutaneous fat and 298 g in visceral fat. The points of greatest loss have been pointed out. The left figure indicates before and the right indicates after.

The changes in MRI images and fat values for a male and female patient are presented in Figures 4 and 5.

## 7 Side Effects

No side effects were observed, except in some patients, a slight redness in the treated area that disappeared within 20 min. The low levels of C‐reactive protein confirm that there are no inflammatory effects, values that are maintained after 6 months.

## 8 Discussion

After the 10 treatment sessions with 30 min of intervention with the device based on amplified energy applied with a belt adapted to the abdominal area through an energy delivery area of 800cm^2^, followed by 45 min of aerobic exercise so that the muscle consumed the triglycerides released, there was a statistically significant decrease in VAT throughout the abdomen. The specific fat loss per session remains undetermined; however, it is evident that the daily application of continuous sessions enhances the reduction of visceral adipose tissue (VAT), as demonstrated by the MRI results at the end of the treatment period.

Regarding the InBody measurements, the mean values of body fat mass decreased from 36.21 to 35.07 (*p* = 0.002), while the percentage of body fat remained unchanged, from 38.52 to 38.22 (*p* = 0.334). These findings contrast with the results obtained from the MRI fat quantification, which yielded different outcomes. These results are consistent with the findings of Chaudry et al., who suggest that impedance measurements are more strongly correlated with visceral fat than with abdominal fat [17].

Regarding the effect of increased energy on adipose tissue and fat loss, it has been reported that temperature increases at a depth of 7–12 mm at 50°C and can reach temperatures of 45°C–55°C [2]. This temperature is what is needed for the fat inside the adipocyte to dissolve. The heating of adipose tissue is selective, inducing thermal damage to SATs, in our opinion, apoptosis, because it is not accompanied by an inflammatory process, as evidenced by the CRP. In another study with other devices, it has been pointed out that, in biopsies, adipocytes in the subcutaneous tissue show signs of membrane injury and changes that could be indicated as changes related to incipient necrosis, in addition to a reduction in the mass of fatty tissue, when accompanied by inflammation. Recently, it has been reported that RF increases the apoptotic index in adipocytes after 1 h of treatment. In this study, the temperature is increased to 45°C in the fat layer, while the temperature at the surface of the skin is much lower [18].

One of the effects that can be observed in this study is the decrease in VAT in the subjects studied. The depth of penetration of energy through the area and power produces an average loss of 430 cm^3^. These results contrast with those carried out by Keating et al. (2015) [19], who, in light to moderate exercise, four times a week for 8 weeks and for 60 min, reported a loss of 287 cm³ of visceral fat measured by MRI. These results are less than half of those obtained in this research, in just 12 days with the application of specialized technology for abdominal fat.

On the other hand, Puig et al. 2022 analyzed the effectiveness of an intervention in reducing VAT in two patients using a device with a low power of 448 KHz and electrodes manually applied, which have a smaller coverage area and dissipate more heat [20]. They found an increase of 2.1% in VAT in one patient and a decrease of 8.54% in the second one, indicating the effectiveness of the studied device for VAT loss. They evaluated this as a favorable method for the reduction of the VAT. This allows us to affirm that the research carried out with the study device, with an abdominal belt with static electrodes with a surface area of 800 cm^2^, with temperature sensors, is a recommended method for VAT loss.

The amount of energy introduced into the subjects during the procedure of application of the energy‐based device and the short time of its application suggest that there is an increase in lipids in the bloodstream, which can accelerate underlying processes in the patient. Hence, it is proposed to introduce a submaximal exercise at the end of the adipocyte lipid output to increase beta‐oxidation. To enhance beta‐oxidation, it was decided to maintain exercise for 45 min at an intensity between 45% and 55% of RHR to ensure lipid oxidation [21] in the large masses of muscle groups involved during exercise. Therefore, the implementation of high‐intensity RF resulted in a significant reduction in abdominal adipose tissue, encompassing both visceral and subcutaneous fat layers. To counteract the elevation of circulating lipids induced by lipolysis, a 45‐min aerobic exercise regimen was introduced, effectively promoting mitochondrial β‐oxidation and maintaining plasma lipid equilibrium.

In other studies, it has been pointed out that it does not decrease BMI because it is a local treatment [22]. In our study, it was found that there was a decrease in body weight and, consequently, also in BMI. The reduction in BMI is statistically significant, so it achieves not only a local effect but also the whole body.

Analysis of subcutaneous and visceral fat after 6 months of treatment: this low retention rate can be attributed to several factors, such as the time burden, especially the long and uncomfortable MRI scans, improvement in health status, and the lack of perceived benefit for the patient and for the research. However, the long‐term effect shows that fat loss continues beyond the 10‐day intervention, as neither patient increased SAT or VAT. On the contrary, fat loss continued, with these results being statistically significant. If closer monitoring had been used, better, more continuous, and closer monitoring methods would have resulted in greater participant adherence [23].

Long‐term effects have already been reported in the use of electromagnetic fields (EMFs) in the treatment of pain and in increasing back mobility in patients with low back pain [24]. In another study, it is noted that pulsed electromagnetic field (PEMF) results in an increase in the beneficial effects of bone markers in osteoporosis [25]. Although a VAT reduction effect had not been reported in adipocytes, according to the results of our study, it seems that they can also be observed in the long term in body fat. However, more research is required to corroborate and explain this effect.

There has been a notable decline in total serum lipids, as shown in a study that examined coalworkers with durations of over 10 years [26]. While the specific mechanisms behind this occurrence are currently insufficiently understood, research has suggested that the main contributor to this phenomenon is Ca^2+^. This ion is also thought to be involved in the apoptosis process in lipid‐filled cells, thereby potentially explaining the persistent consequences of this phenomenon. Additionally, previous studies have demonstrated the significant role of Ca^2+^ concerning regulatory cell death in interactions between the endoplasmic reticulum and mitochondria. Collectively, these findings provide a possible explanation for the continued decline in adipose tissue over a prolonged period [27].

HDL and LDL values are not significant because abdominal fat has cholesterol in free form that is not esterified, which is transported as lipoproteins [28]. In addition, exercise training alone has shown no significant effect on LDL‐C and little HDL [28].

A limitation of this study is the lack of a control group for direct comparison, primarily due to practical constraints such as limited resources and the ethical challenge of withholding treatment from a control group. Participants were instructed to refrain from altering their lifestyle, including diet and exercise, during the study period. While the absence of a control group restricts the ability to draw definitive conclusions, the effect of the treatment has already been studied in 20 patients in Clinical Trial NCT06377358, which allows us to give value to the results obtained. Investigator‐sponsored studies, in contrast to industry‐sponsored trials, are often subject to higher dropout rates, driven by limited human and financial resources [26]. To mitigate this issue, future follow‐up studies will implement strict guidelines to reduce participant dropout, and expanding the sample size may further address challenges related to patient retention and study power. The objective of incorporating exercise to reduce the plasma concentration of triglycerides has been met since there is a statistically significant decrease, thus allowing the conclusion that when exercise is added to the treatment, there is no mobilization of triglycerides in the blood.

The possibility of conducting a randomized controlled trial (RCT) in future research could enhance the generalizability and robustness of the findings [29].

## 9 Conclusion

After 10 consecutive treatment sessions (excluding weekends), the use of a specialized device with an abdominal belt containing static electrodes (800 cm^2^ surface area) and temperature sensors resulted in a significant reduction in both subcutaneous and visceral abdominal fat, as well as improvements in body weight, waist‐hip ratio, and BMI. The addition of exercise further reduced blood triglyceride levels, indicating enhanced triglyceride metabolism.

These findings suggest that combining the device with regular exercise may effectively reduce abdominal adipose tissue and improve plasma lipid levels. Follow‐up at 6 months showed continued fat loss, with no restoration of subcutaneous or visceral fat. This intervention demonstrates efficacy and safety in reducing metabolic risk factors, including waist‐to‐hip ratio, insulin levels, and BMI.

## Ethics Statement

This study was approved by the Central Ethics Committee of the Universitat Autònoma de Barcelona (Reference Number: CEEAH CA31) and conducted in accordance with the principles outlined in the Declaration of Helsinki.

## Consent

All participants provided written informed consent prior to their inclusion in the study, following full disclosure of the research aims, procedures, and potential risks.

## Conflicts of Interest

The authors declare no conflicts of interest.

## Data Availability

The data that support the findings of this study are available from the corresponding author upon reasonable request.
